# Lessons learned from a double-blind randomised placebo-controlled study with a iota-carrageenan nasal spray as medical device in children with acute symptoms of common cold

**DOI:** 10.1186/1472-6882-12-147

**Published:** 2012-09-05

**Authors:** Tamas Fazekas, Philipp Eickhoff, Nathalie Pruckner, Georg Vollnhofer, Gustav Fischmeister, Christopher Diakos, Margit Rauch, Maria Verdianz, Andreas Zoubek, Helmut Gadner, Thomas Lion

**Affiliations:** 1 St. Anna Children’s Hospital, Vienna, Austria; 2 Children’s Cancer Research Institute and LabDia Labordiagnostik, Vienna, Austria

**Keywords:** Carrageenan, Common cold, Viral infection, Upper respiratory tract infections, Pediatric pulmonology

## Abstract

**Background:**

Common cold is caused by a variety of respiratory viruses. The prevalence in children is high, and it potentially contributes to significant morbidity. Iota-carragenan, a polymer derived from red seaweed, has reduced viral load in nasal secretions and alleviated symptoms in adults with common cold.

**Methods:**

We have assessed the antiviral and therapeutic activity of a nasal spray containing iota-carrageenan in children with acute symptoms of common cold. A cohort of 153 children between 1–18 years (mean age 5 years), displaying acute symptoms of common cold were randomly assigned to treatment with a nasal spray containing iota-carrageenan (0.12%) as verum or 0.9% sodium chloride solution as placebo for seven days. Symptoms of common cold were recorded and the viral load of respiratory viruses in nasal secretions was determined at two consecutive visits.

**Results:**

The results of the present study showed no significant difference between the iota carrageenan and the placebo group on the mean of TSS between study days 2–7. Secondary endpoints, such as reduced time to clearance of disease (7.6 vs 9.4 days; p = 0.038), reduction of viral load (p = 0.026), and lower incidence of secondary infections with other respiratory viruses (p = 0.046) indicated beneficial effects of iota-carrageenan in this population. The treatment was safe and well tolerated, with less side effects observed in the verum group compared to placebo.

**Conclusion:**

In this study iota-carrageenan did not alleviate symptoms in children with acute symptoms of common cold, but significantly reduced viral load in nasal secretions that may have important implications for future studies.

**Trial registration:**

ISRCTN52519535, http://www.controlled-trials.com/ISRCTN52519535/

## Background

Acute viral infection of the upper respiratory tract, also referred to as common cold, is the most frequently observed disease in humans. Respiratory viral infections lead to more than 400.000 hospitalizations per year in children below 18 years of age in the United States [1]. The considerable morbidity and mortality in adults and infants caused by respiratory viral infections has recently been reviewed [2,3]. The morbidity caused by viral upper respiratory tract infections (URTI) and the ensuing complications are more pronounced in individuals with pre-existing respiratory conditions such as asthma [4,5]. While numerous treatment approaches have been claimed to reduce symptoms [6], no interventions were conclusively demonstrated to display antiviral activity, and to effectively decrease the duration or severity of manifestations. A recent review reported the lack of efficacy of OTC (over-the-counter) cough and cold medicines in children, and revealed their inability to reduce the rate of severe adverse events [7]. Substances such as diphenhydramine and codeine have been associated with significant side effects in children, thus restricting the applicability of several currently available therapies [8]. Recently, a potent antiviral effect against several respiratory viruses was demonstrated for iota-carrageenan, a polymer derived from red seaweed [9,10]. Carrageenan has been shown to display antiviral activity against a range of animal viruses [11] and has been tested clinically for prevention of sexually transmitted HIV-1 viral infections [12,13]. Iota-carrageenan has recently been shown to be a potent inhibitor of papilloma virus *in-vitro*, even at concentrations below 1 μg/ml [14]. Carrageenan has been used as food additive for centuries. As an indigestible polysaccharide extracted from red algae (seaweed), it was added to foods as a gelling agent or emulsifier. In 1959, carrageenan was granted GRAS (Generally Recognized as Safe) status in the United States, thus documenting the safety of this substance. Carrageenan increases the viscosity if applied as nasal spray, thereby prolonging the humidification of the nasal mucosa. In addition, the solution forms a barrier by direct interaction of carrageenan with viruses, such as human rhinoviruses, which are trapped and inactivated by the polymer. Results of a recent pilot study in 35 adult patients showed that application of a nasal spray containing iota-carrageenan three times per day alleviated symptoms of common cold and reduced the viral load in the nasal mucosa. Hereby, the efficacy of Carrageenan was shown on local symptoms, whereas systemic symptoms remained the same [15].

Based on these observations, the present study was conducted to evaluate the antiviral efficacy of a nasal spray containing iota-carrageenan in children, with regard to alleviation of symptoms and sequelae of common cold.

## Methods

A randomized, double blind placebo-controlled pilot study was conducted in two study centers, the St. Anna Children’s Hospital and a private pediatric clinic in Vienna, Austria.

### Total Symptom Score (TSS)

The TSS used was based on the evaluation of eight leading clinical features including the systemic symptoms headache, muscle ache, chilliness (systemic symptom score, SSS), and the local symptoms including sore throat, blocked nose, runny nose, cough, and sneezing (local symptom score, LSS), according to a published scoring system [16]. Severity was rated on a four-point scale with zero indicating the absence of symptoms, and scores of 1 to 3 representing mild, moderate and severe symptoms, respectively.

### Patients

Previously healthy, immunocompetent children and adolescents between 1 and 18 years of age with symptoms of acute rhinitis prevailing for less than 48 hours, and a total symptom score (TSS) ≤9, were enrolled in the study between January 2009 and January 2010. This low symptom score of 9 was chosen as inclusion criterion because a higher TSS would have led to the use of more co-medications, potentially biasing the effect of Carrageenan. Immunocompetence was evaluated clinically, including a history of less than 5 bacterial infections per year without a stay on intensive care units and without a history of any chronic disease. URTI was defined by the presence of appropriate symptoms according to a scale described above. Suspected bacterial infections, recent use of antimicrobial drugs, and intranasal treatment at first presentation were exclusion criteria. However, additional medication could be prescribed in case of fever, bacterial otitis, wheezing, persistent coughing or pneumonia upon decision of the physician. In line with the Austrian legal requirements, written informed consent was obtained from children above 14 years of age and from their legal guardians in all instances.

### Study Procedures

At enrolment into the study (first visit), medical history was recorded, physical examination was performed, and samples for molecular virus testing were obtained by a standardized aspiration of nasal secretions. The samples were frozen and stored at −80°C until use. Patients were assigned to receive a nasal spray containing either iota-carrageenan (0.12% / 0.5% sodium chloride) or placebo (0.9% sodium chloride) in a randomized and blinded manner (allocation ratio 1:1), using simple (unrestricted) randomisation. The concentration of iota-carrageenan and sodium chloride for the study medication was chosen identically compared to the previously performed pilot study [15], using the optimal galenic composition. The nasal spray containers were sequentially numbered. The random allocation sequence was generated by an independent statistician, the participants were enrolled by a study nurse and the investigating pediatricians assigned participants to the interventions. The children and/or their legal guardians received a standardized diary for the recording of symptoms and additional medication. A single puff (0.14 ml) of the spray per nostril was to be administered three times per day for a total of seven days. The patients and parents were requested to record symptoms permitting assessment of the TSS during the first week of treatment. Over the following two weeks (days 8 to 21), recording of the presence or absence of any symptoms was requested for determination of disease duration. Follow-up examination of the patients and collection of nasal secretion samples for molecular virus testing were performed three to five days into treatment (second visit). Final examination and collection of symptom diaries for the evaluation were carried out on the third visit after day 21. Molecular screening and quantitative assessment of viral load were performed for respiratory viruses including rhinovirus (RV), respiratory syncytial virus (RSV), human metapneumovirus (MPV), influenza A (InfA) virus, influenza B (InfB) virus, parainfluenzavirus (PIV1, PIV2, PIV3), and coronaviruses (CoV) by methods described previously [17,18] (for more detailed description of virus analysis see Additional file 1).

### Ethics Statement

The study has been approved by the local IRB Committee named Ethikkommission St. Anna Kinderspital (project approval number 190109). In line with the Austrian legal requirements, written informed consent was obtained from children above 14 years of age and from their legal guardians in all instances. The study was performed in compliance with the ICH E6 Note for Guidance on Good Clinical Practices (CPMP/ICH/135/95)5, the principles of the Declaration of Helsinki, local drug regulations, and standard operating procedures of the investigator, sponsor and CROs involved. The study protocol and attached documentation were approved by the responsible Ethics Committee.

### Statistics

Comparison between the verum and placebo groups was done by means of Mann–Whitney U-tests for continuous variables and by means of Chi square tests for ordinal or nominal variables. The viral load was compared using Student’s T-test after logarithmic transformation of the data. For comparison of time to disease clearance, the Log Rank (Mantel Cox) test was used. Calculation of the required sample size was based on previous results obtained in adult patients [15], which indicated that data from 70 individuals would be sufficient to obtain significant results on the primary endpoint, the mean of TSS on days 2 to 7 at the 5% significance level and 80 percent power. It was decided to recruit at least 200 patients in order to be able to analyze an ITT (intention to treat) population of at least 150 patients, with further analysis of per protocol (PP) patients.

## Results

### Viral pathogens detected in the patient cohorts investigated

**Figure 1:**
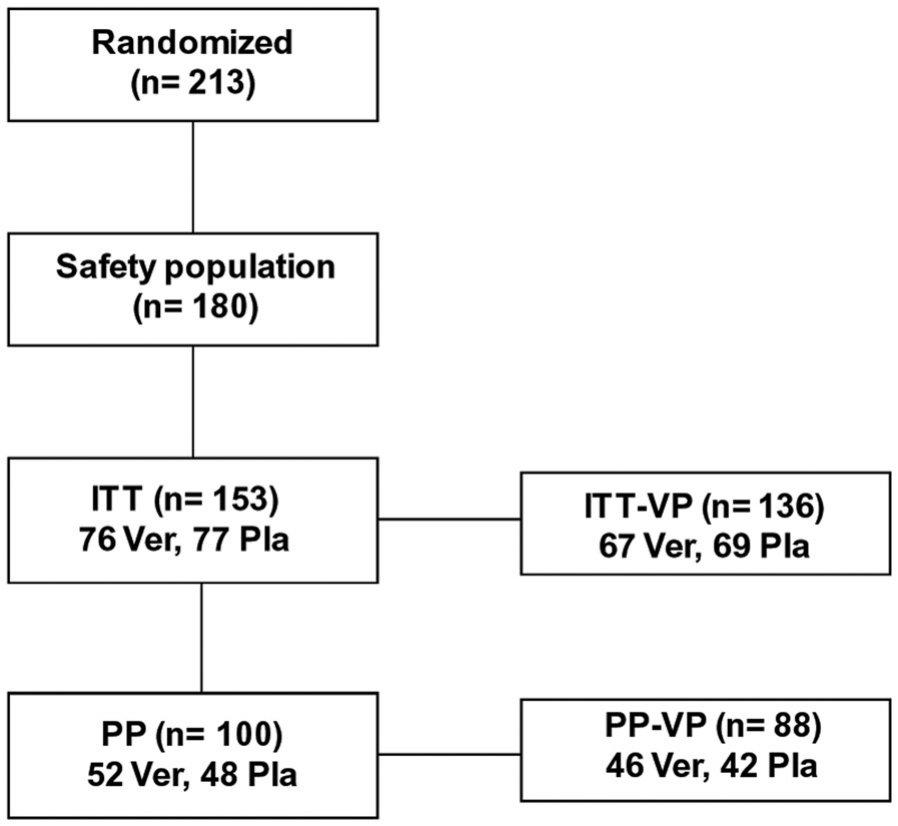
**Virus Distribution.** The distribution of respiratory viruses is shown in percent of total positive specimens. Parainfluenzavirus includes subtypes 1–3; Coronavirus includes subtypes 229E and OC43.

**Figure 2:**
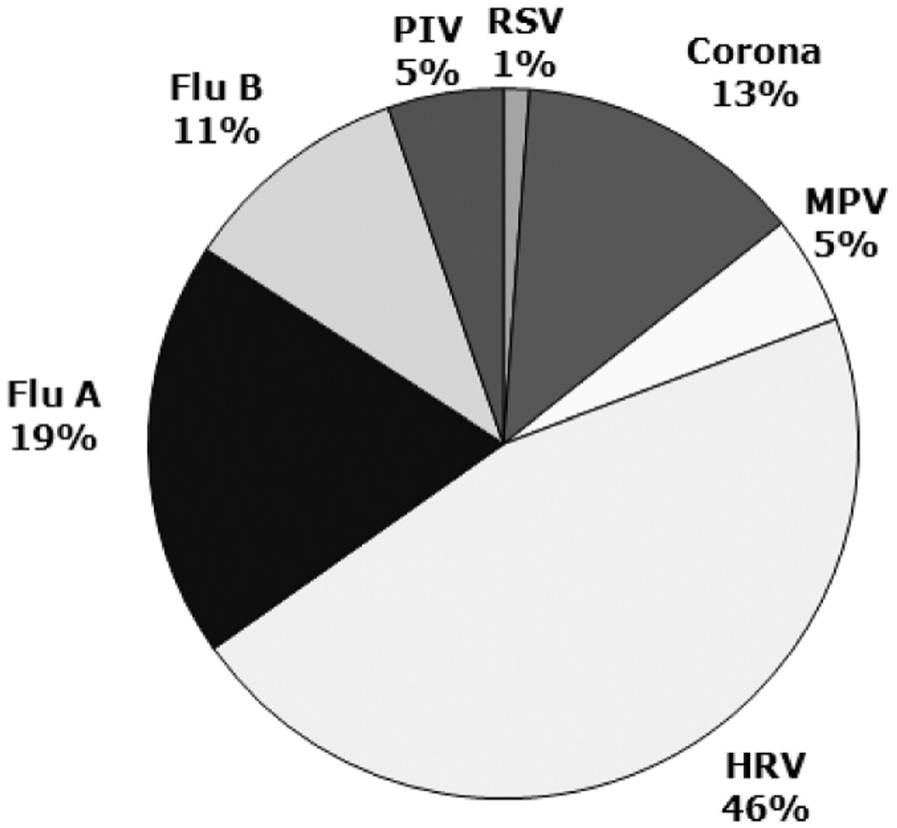
Symptom scores in the ITT and PP cohort.

A total of 213 patients with clinically confirmed acute symptoms of common cold were assessed for eligibility within a one year study period (Figure 1). Patients` characteristics are shown in Table 1. The main reason for exclusion was the failure to return the study documents, and in a few cases withdrawal of consent. Ultimately, 153 children could be included in the ITT cohort, and were analyzed accordingly. The majority of children (88.8%) tested positive for at least one virus (mean 1.5; range 1–5) at visits 1 or 2. The most frequently detected viral pathogen was human rhinovirus (46%) followed by coronavirus (13%), influenza A virus (19%), influenza B virus (11%), human metapneumavirus (5%), parainfluenzavirus (5%) and respiratory syncytial virus (1%). The number of viruses detected in individual patient samples obtained at visits one and two in both groups is displayed in Figure 2.

**Table 1:** Characteristics of 213 pediatric patients with acute symptoms of common cold

**Characteristic**	**Placebo n=104**	**Iota-Carrageenan=109**
Mean age-years (std deviation)	5.3 (3.6)	4.6 (3.6)
Mean weight-kilograms (std deviation)	22.6 (12.2)	20 (12)
Mean height- centimeters (std deviation)	112.7 (24.1)	108.3 (24.7)
Male gender-total (%)	42 (40.4)	62 (56.9)
Body-Mass Index (std deviation)	17.3 (3.0)	17.3 (3.3)
Smoke exposure-total (%)	15 (14.4)	11 (10.2)

### Clinical efficacy and safety

**Figure 3:**
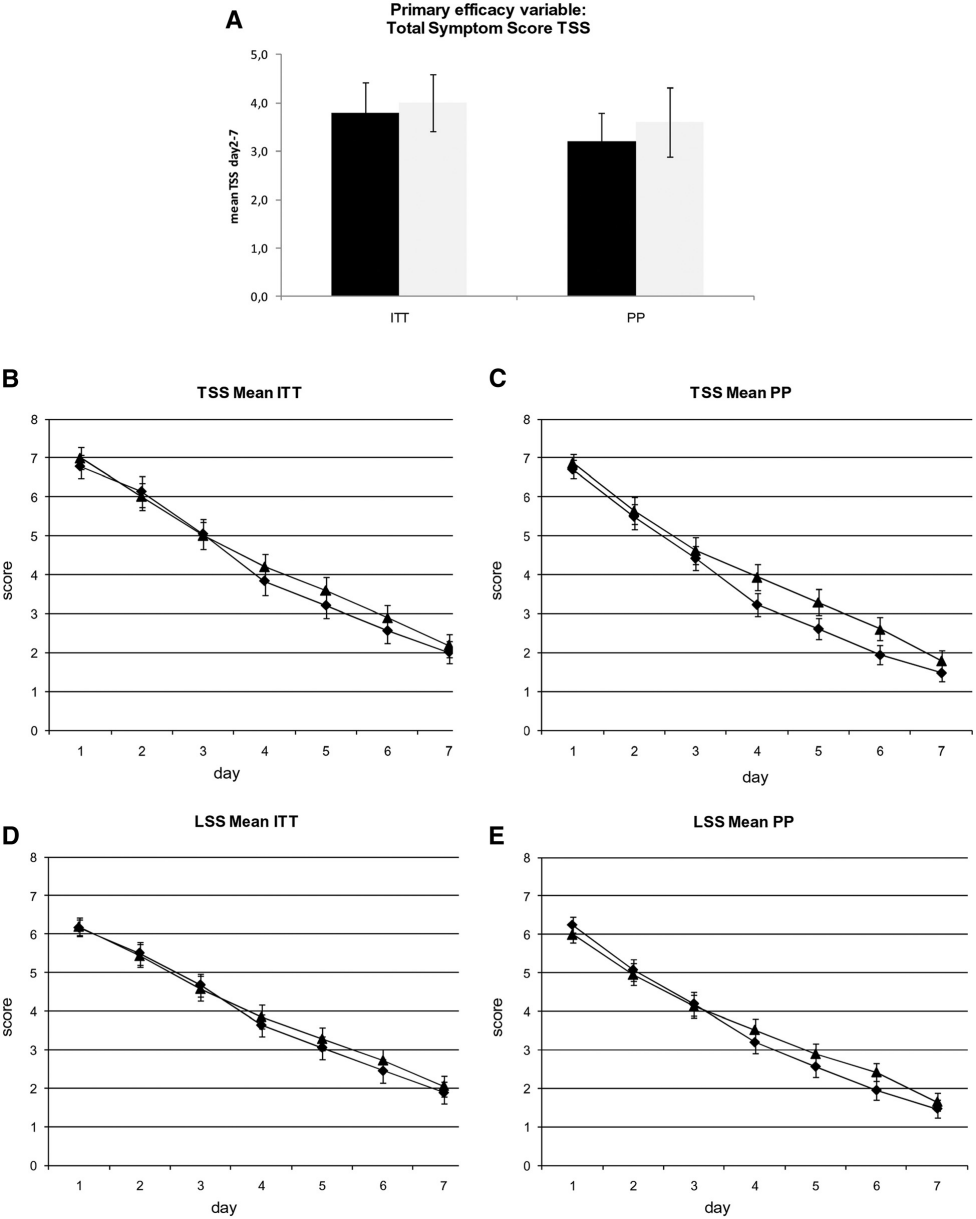
Enrollment and randomization scheme.

In the 153 patients of the ITT cohort, no significant differences were observed between verum and placebo treatment with regard to the primary efficacy variable, the mean of total symptom scores (TSS) on days 2 to 7 of the study (Figure 3, Additional file 2: Table S1). The mean TSS between days 2–7 was 3.8 (SD 2.8) in the iota-carrageenan group and 4.0 (SD 2.6) in the placebo group. Pre-defined secondary endpoints including TSS on different study days, total systemic and total local symptom scores also showed no significant differences between the iota-carrageenan and the placebo group (Figure 3B-E).

**Figure 4:**
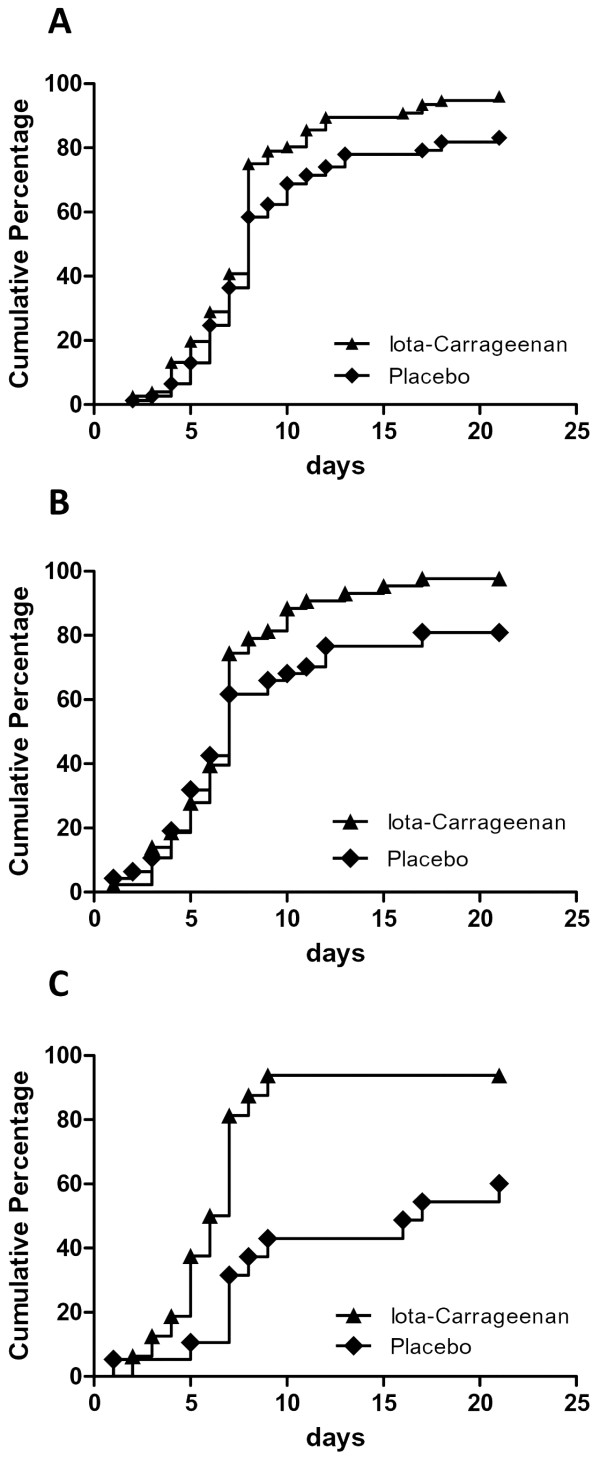
**Alleviation of symptoms in patients with early symptoms of common cold treated either with iota-carrageenan nasal spray or placebo nasal spray.** Alleviation was defined as persistent disappearance of symptoms during the observation period. Patients with recurrent disease were rated as symptomatic until complete resolution of symptoms. (**A**) Cumulative percentage of patients from the ITT population with complete persistent alleviation of symptoms (p < 0.038). (**B**) Rhinovirus-positive patients (n.s. p = 0.06). (**C**) Coronavirus-positive patients (p < 0.002). P-values were determined by the Log-rank (Mantel-Cox) test.

Detailed symptom-diaries were only requested during the first week of the study. Between days 8–21, only the presence or absence of common cold symptoms had to be recorded. At the end of the study period of 21 days, a significantly higher proportion of patients receiving placebo (12/77; 15.6%) reported symptoms in comparison to only 3/77 (3.9%) patients in the group treated by verum (p < 0.016). Moreover, the mean time to persistent symptom alleviation reported by patients in the verum-treated group was significantly shorter in comparison to the placebo group (7.6 versus 9.4 days; p = 0.038) (Figure 4A). A post hoc analysis in patients with the most commonly observed viral pathogens including rhinovirus (n = 90) and coronavirus (n = 35) revealed a similar advantage for the verum-treated group (Figures 4B and 4C).

The use of the nasal sprays was well tolerated. The overall frequency of drug-related adverse events tended to be lower in the iota-carrageenan group compared to placebo. In the study period, adverse events related to the upper respiratory tract were most common. Treatment-unrelated severe adverse events (SAEs) were observed in four patients, including two in the iota-carrageenan group and two in the placebo group.

### Monitoring of viral load and secondary infections

**Figure 5:**
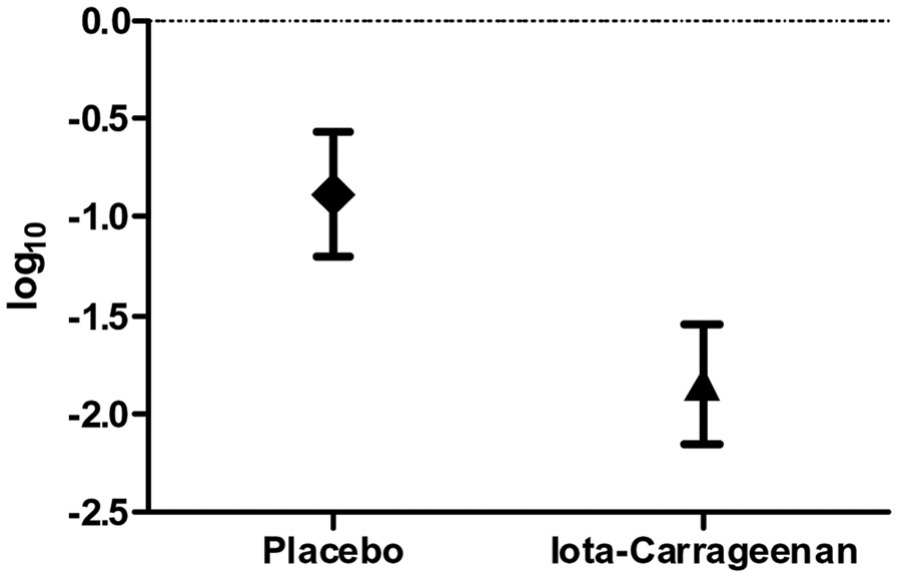
**Reduction of viral load between visits 1 and 2.** The log_10_ of the virus copies was determined by quantitative PCR analysis, and the difference in viral load between visits was calculated for the verum and the placebo group. The log_10_ difference in total virus copy number (±SEM) between visits 1 and 2 is shown (p < 0.026).

A secondary objective of the study was to investigate the effect of iota-carrageenan nasal spray on virus elimination in nasal fluid samples. The incidence of viruses observed was typical for common cold (Figure 2). Topical application of the iota-carrageenan spray reduced viral loads in nasal secretions to a significantly higher degree than placebo after 3 to 5 days of treatment (p = 0.026; Figure 5), and the documented reduction in the number of viruses between visits 1 and 2 was significantly greater in the verum group. The antiviral efficacy of iota-carrageenan is further supported by the fact that 27% of patients in the verum group and less than 13% in the placebo group tested virus-negative at the second visit (Additional file 3: Figure S1B). The observed decrease in virus-positive patients between visits 1 and 2 was statistically significant only in the verum group (p < 0.001 versus p = 0.804). At the second visit, the mean number of viruses detected in the iota-carrageenan group was reduced from 1.3 to 0.8 (p < 0.001), while in the placebo group the observed reduction from 1.3 to 1.1 was not significant. Moreover, 52.2% of verum patients and 32.4% placebo patients had cleared their viruses by the second visit (p = 0.030). At visit 2, the incidence of newly acquired infections with viruses that had not been detected at visit 1 was significantly lower in the verum group (13% versus 27%; p = 0.046).

### Use of co-medication

Most patients (90%) received at least one additional drug compatible with the study protocol as concomitant medication, including non-steroidal anti-inflammatory agents (64%), antitussives (57%), antibiotics (23%), ß2-sympathomimetics (23%), and others (12%) (Additional file 3: Figure S1). To address the notion that less co-medication could permit better appreciation of the effect of iota-carrageenan, a post hoc analysis was conducted in a subgroup of patients (n = 51) who received either no co-medication (n = 14) or one additional drug only (n = 37). Patients receiving verum showed a trend towards lower TSS compared to placebo-treated patients (Additional file 3: Figure S1B).

## Discussion

The need for therapeutic strategies against URTI in children is highlighted by results of randomized studies on the use of OTC preparations for treatment of common cold in children below 12 years of age, which revealed no significant differences between verum and placebo [19]. Due to insufficient data on the application of nasal decongestants for common cold in children, their use is not recommended in patients below 12 years of age [20]. Upper respiratory tract infections in early childhood can contribute to significant morbidity resulting from respiratory complications even in immunocompetent individuals. To date, no causal therapy is available in most instances, because URTIs are caused predominantly by viral pathogens for which no effective therapeutic agents have been available.

The results of the present study showed no significant difference between the iota-carrageenan and the placebo group on the predefined primary endpoint, the mean of TSS between study days 2–7. This may be due to the low TSS level below 10 at inclusion, which was necessary to avoid a bias by a higher use of co-medications. Furthermore, the concentration of iota-carrageenan which was the same as used in the previous pilot study in adults [15], may also have an influence on symptom scores. About 90% of children in both study groups received one or more co-medications. In the verum group, predominantly antiinflammatory agents (58%) or antitussives (62%), and less frequently antibiotics (27%) or ß2-sympathomimetics (26%) were prescribed. It is conceivable that the systemic and the local symptom scores were respectively affected by the antipyretic and the antitussive medication; hence negative effects of the unusually high prevalence of patients with multiple co-medications may have masked the effect of the study medication.

The age of the patients in our study ranged between one and 18 years, with a mean of five years. In young children, it is often very difficult to evaluate symptoms like headache, muscle ache or chilliness. These difficulties in evaluating symptoms in children were recently discussed by Taylor et al. [21], and require careful consideration in future studies in pediatric patient cohorts.

However, the mean difference in time to complete symptom alleviation between the verum and the placebo group was 1.8 days, while the reported effect of neuraminidase inhibitors on the treatment of influenza in adults showed an overall benefit over placebo treatment of approximately one day [22].

Another secondary objective of the study was determination of the viral pathogens and intranasal viral loads. Recent data demonstrated that specific viral infections and co-infections as well as viral load contribute to disease severity in children with respiratory infections [23-25]. The viruses detected in our study were similar to earlier reports, and did not reveal constellations associated with a particularly high risk of complications. Significantly more children in the verum group tested virus-negative at visit 2, and fewer children displayed multiple viruses or revealed a new viral infection at this time. These findings may have important implications. Recurrent viral episodes prolonging reactive bronchial inflammation are frequently encountered complications of URTI. In this regard, the topical effect of iota-carrageenan could be instrumental by reducing the incidence of subsequent lower respiratory tract involvement.

The major limitations of this study include the restricted performance of no more than two nasal aspirations, because newly acquired viral infections after day 3–5 could have occurred in the placebo group during the follow up period, potentially changing the proportion of patients with no symptoms. A second limitation may be given by the TSS questionnaire, which may not be the most appropriate for a study population with a mean age of 5 years.

Despite the fact that viral load in nasal secretions were reduced by iota-carrageenan, no significant alleviation of symptoms in children with common cold could be observed in this prospective, randomized, placebo-controlled pediatric study.

## Competing interests

Funding from Marinomed Biotechnologie GmbH for the logistics of virus sampling for the laboratory where the authors TL, MR and MV work. All other authors declare no conflict of interests or any financial and personal relationships with other people or organisations that could inappropriately influence their work.

## Authors’ contributions

Conception and design of the study was contributed by TF, AZ; data analysis and interpretation was performed by PE, NP, MR, MV; drafting the manuscript for important intellectual content was made by HW, GV, GF, CD, HG, TL. All authors read and approved the final manuscript.

## What is already known on this topic

In adults, nasal sprays with Carrageenan significantly reduce symptoms of common cold.

## What this study adds

In this first pediatric study, Carrageenan significantly reduces viral load and cytokine levels in nasal mucosa of children.

## Funding statement

The authors acknowledge Marinomed Biotechnologie GmbH, Vienna, Austria, who provided financial support for this study (grant number COP0109). The funder has supported a part of the logistics. The sponsors had no influence on study design, collection, analysis, and interpretation of data.

## Pre-publication history

The pre-publication history for this paper can be accessed here:

http://www.biomedcentral.com/1472-6882/12/147/prepub

## Supplementary Material

Additional file 1 **Methodology and spectrum of virus analysis.** Detailed description of RQ-PCR, including unpublished primer and probes. Click here for file

Additional file 2: Table S1 Summary of primary and secondary outcome measures. The summary of primary and secondary outcome measures is shown. ITT (intention to treat), PP (per protocol), TSS (total symptom score), SSS (systemic symptom score), LSS (local symptom score). Click here for file

Additional file 3: Figure S1 Co-medications and total symptom scores (TSS) in the study cohort and the placebo cohort. Click here for file
