# Supplementary material for: Lessons learned from a double-blind randomised placebo-controlled study with a iota-carrageenan nasal spray as medical device in children with acute symptoms of common cold
Source: BMC Complement Altern Med. 2012 Sep 5;12:147. doi: 10.1186/1472-6882-12-147 (PMC3575307; doi:10.1186/1472-6882-12-147)
Supplement: Additional file 1 — Methodology and spectrum of virus analysis. Detailed description of RQ-PCR, including unpublished primer and probes. [file 1472-6882-12-147-S1.doc]

**Methodology and spectrum of virus analysis**

Clinical specimens were transferred to the laboratory immediately after sampling. The specimens were then quick-frozen in 140 µl aliquots and stored at -80°C until analysis. Prior to RNA isolation, the specimens were spiked with defined amounts of a control virus (the Phocine Distemper Virus-PDV), which served as an internal control for efficient nucleic acid extraction and reverse transcription. Nucleic acid extraction of patient specimens spiked with control virus was performed with the Qiagen-Kit (Qiagen, Hilden, Germany QIAamp Viral RNA Kit (52906). For detection of RNA viruses, a reverse transcription step was performed to generate cDNA using the M-MLV Reverse Transcriptase (Promega, Madison, USA). Reverse transcription was carried out for one hour at 37 °C.

The quantitative RQ-PCR reactions were carried out in duplicates on a Roche Light Cycler 480 using hydrolysis probe assays. Briefly, typical reactions contained 10 µl of 2xProbes Master (Roche Diagnostics, Mannheim, Germany), 300 nM of each primer, 100 nM of each probe and 5 µl sample in a total volume of 20 µl. Cycling conditions were as follows: 1 min 50 °C and 10 min at 95 °C, followed by 50 cycles each consisting of 15 s at 95 °C and 1 min at 60 °C. Data were analyzed using the LC480 software.

Molecular screening was carried out for the 6 most common RNA viruses known to cause upper and lower respiratory tract infections in immunocompromised individuals including the Respiratory Syncytial Virus (RSV), Rhinovirus (RV), human Metapneumovirus (MPV), Influenza A (InfA) virus, Influenza B (InfB) virus, Coronavirus (CoV; (229E and OC43)) and Parainfluenzavirus (PIV1, PIV2, PIV3) (2, 7, 10, 19, 20, 21, 22). The assays for the detection of RSV, InfA, PIV1/2/3 are described in detail in our pertinent publications [22, 24],

Unpublished primer and probes were as follows (5´-3´): hMPV_B forward GCTGAGCAATCAAGAGAATGCA, reverse GCACCGAGAGGTGATAGTGCA, probe FAM-CCCATGCAAAGTCAGCACAGGAAGACAC-TAMRA; hMPV_A forward GCTGAGCAATCAAAGGAGTGC, reverse GCAACCAGAGCCCCRAGAG, probe FAM-CACAGGAAGGCATCCTATCAGTATGGTTGC-TAMRA, RV forward 1b CTAGTTTGGTCGATGAGGCTAGGA, forward 2b CTAGTCTGGTCGATGAGGCTGAG, forward 3a AGACCTGGCAGATGAGGCTRGA, reverse1 CACATTCAGGGGCCGGA, reverse2 CGCATTCAGGGGCCG, probe MGBa FAM-CCTGCGTGGCTGC-NFQ, probe MGBb FAM-CCTGCGTGGCGGCC-NFQ; InfB forward ARACCAGAGGGAAACTATGCC , InfB reverse YCCRGATGTAACAGGTCTGACT, InfB K probe FAM-ACCTTCGGCAAAAGCTTCAATACTCCA-TAMRA, InfB M probe FAM-ACCCTCGGCAAGAGTTTCAATACTCCA-TAMRA; PDV forward CGGGTGCCTTTTACAAGAAC, reverse TTCTTTCCTCAACCTCGTCC and probe YY-ATGCAAGGGCCAATTCTTCCAAGTT-TAMRA. Each PCR assay contained a number of additional controls including virus-specific positive controls and negative controls. In parallel, RQ-PCR analysis of the spiked control virus was performed. For eligible results, the positive controls had to be within a specified range of CP values and all negative controls had to reveal negative results.

**Thomas Lion, MD, PhD**

Professor and Medical Director

**Labdia Labordiagnostik GmbH**

CHILDREN'S CANCER RESEARCH INSTITUTE

St. Anna Kinderkrebsforschung

Zimmermannplatz 8, 1090 Vienna, Austria

T +43(1)40077-4890 (Secretary: -4800)

F +43(1)40077-64890

thomas.lion@ccri.at

[thomas.lion@ccri.at](mailto:thomas.lion@ccri.at) [www.labdia.at](http://www.labdia.at/)
