# Supplementary material for: Lessons learned from a double-blind randomised placebo-controlled study with a iota-carrageenan nasal spray as medical device in children with acute symptoms of common cold
Source: BMC Complement Altern Med. 2012 Sep 5;12:147. doi: 10.1186/1472-6882-12-147 (PMC3575307; doi:10.1186/1472-6882-12-147)
Supplement: Additional file 2: Table S1 — Summary of primary and secondary outcome measures. The summary of primary and secondary outcome measures is shown. ITT (intention to treat), PP (per protocol), TSS (total symptom score), SSS (systemic symptom score), LSS (local symptom score). [file 1472-6882-12-147-S2.doc]

**Supplementary Table. Summary of primary and secondary outcome measures**

|  | **ITT Iota-Ca** | **ITT**  **Placebo** | **p** | **PP**  **Iota-Ca** | **PP**  **Placebo** | **p** |
| --- | --- | --- | --- | --- | --- | --- |
| **Primary efficacy variable** TSS mean of days 2-7 | 3.8 (2.8) | 4.0 (2.6) | 0.535 | 3.2 (2.1) | 3.7 (2.5) | 0.446 |
| **Secondary efficacy variable** SSS mean of days 2-7  LSS mean of days 2-7 | 0.3 (0.6)  3.5 (2.5) | 0.3 (0.8)  3.6 (2.3) | 0.811  0.607 | 0.1 (0.3)  3.1 (2.1) | 0.4 (1.0)  3.3 (2.0) | 0.393  0.548 |

Shown is the summary of primary and secondary outcome measures. ITT (intention to treat), PP (per protocol), TSS (total symptom score), SSS (systemic symptom score), LSS (local symptom score).
