# Supplementary figures and images for: Lessons learned from a double-blind randomised placebo-controlled study with a iota-carrageenan nasal spray as medical device in children with acute symptoms of common cold
Source: BMC Complement Altern Med. 2012 Sep 5;12:147. doi: 10.1186/1472-6882-12-147 (PMC3575307; doi:10.1186/1472-6882-12-147)

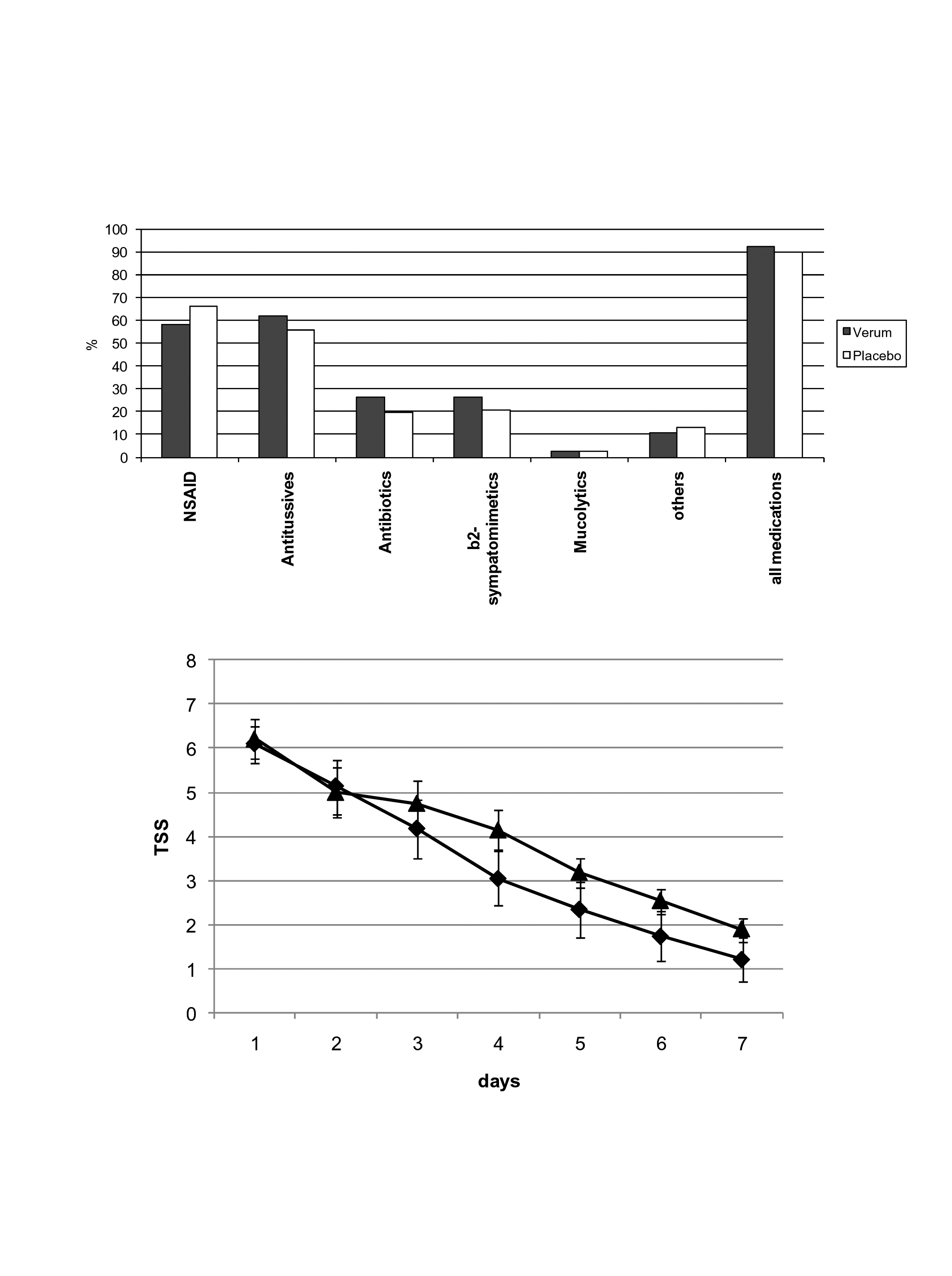

Supplement: Additional file 3: Figure S1 — Co-medications and total symptom scores (TSS) in the study cohort and the placebo cohort. [file 1472-6882-12-147-S3.tiff]
